# Macrophage-Derived Chemokine MDC/CCL22: An Ambiguous Finding in COVID-19

**DOI:** 10.3390/ijms241713083

**Published:** 2023-08-23

**Authors:** Zoia R. Korobova, Natalia A. Arsentieva, Areg A. Totolian

**Affiliations:** 1 Laboratory of Molecular Immunology, Saint Petersburg Pasteur Institute, Mira St. 14, 197101 St. Petersburg, Russia; zoia-korobova@yandex.ru (Z.R.K.); arsentieva_n.a@bk.ru (N.A.A.); 2 Department of Immunology, Pavlov First State Medical University of St. Petersburg, L’va Tolstogo St. 6–8, 197022 St. Petersburg, Russia

**Keywords:** macrophage-derived chemokine, MDC/CCL22, chemokines, novel coronavirus infection, COVID-19, post-COVID

## Abstract

Macrophage-derived chemokine (MDC/CCL22) is a chemokine of the C-C subfamily. It is involved in T-cellular maturation and migration. Our previous research shows that plasma CCL22/MDC tends to show a statistically significant depletion of concentrations in acute patients and convalescents when compared to healthy donors. In the current work, we investigate existing views on MDC/CCL22 dynamics in association with various pathologies, including respiratory diseases and, specifically, COVID-19. Additionally, we present our explanations for the observed decrease in MDC/CCL22 concentrations in COVID-19. The first hypothesis we provide implies that viral products bind to MDC/CCL22 and block its activity. Another explanation for this phenomenon is based on dendritic cells population and the inhibition of their function.

## 1. Introduction

COVID-19 is defined as an acute respiratory infection caused by the RNA-based virus (SARS-CoV-2) of the Betacoronavirus genus. The first cases of COVID-19 were registered in Wuhan, China, in December 2019. By March 2020, the World Health Organization (WHO) had officially named COVID-19 a global pandemic [1,2,3]. As with most viral infections, COVID-19 begins with the virus entering the cell via interactions between viral proteins and complementary receptors on host cellular membranes. SARS-CoV-2 has two ways of entering cells, either through membrane fusion or viral particle uptake. This can activate various innate immune pattern-recognizing receptors (or PRRs), depending on the point of entry. For membrane fusion to occur, the cellular receptors ACE2, TMPRSS2, and sometimes NRP1 are necessary. Once fusion happens, the virus’s genetic material is released into the host cell’s cytoplasm. In COVID-19, angiotensin-converting enzyme 2 (ACE2) receptor usually acts as the major entry point for the virus. It is widely represented in human cells: pneumocytes, enterocytes, vascular endothelial cells, smooth muscle cells, and even glia [4,5,6].

Most SARS-CoV-2 target cells are located in the upper and lower parts of the respiratory system. In addition to inflammation caused by interactions between viral spike protein (S protein) and the ACE2 receptor, there is also the subsequent inhibition of this receptor. This leads to the enhanced expressions of genes responsible for angiotensin II secretion. In turn, angiotensin II binds to Toll-like receptors (type II), which activates inflammatory transcription factors (IRF, IkBɑ, NF-kB). Due to the nuances of COVID-19, such conditions can lead to enhanced inflammation, which can potentially activate a so-called ‘cytokine storm’.

In addition, there is an activation of innate immunity (i.e., DCs, macrophages, granulocytes) that precedes the inclusion of the main effector lymphocytes of adaptive immunity [7,8].

COVID-19 immunity is often followed by the development of a cytokine storm, a pathological hyperinflammatory reaction with increased cytokine secretion that is directly linked with COVID-19 severity. It is often mentioned as the main cause of death in COVID-19 patients [9]. A cytokine storm is often mediated by CC-chemokines, e.g., MCP-1/CCL2, MIP-1α/CCL3, MCP-3/CCL7, and eotaxin-3/CCL26 [9,10], but also by other chemokines. One such chemokine is MDC/CCL22, a promising molecule that can serve as the potential marker of COVID-19 severity.

## 2. Macrophage-Derived Chemokine (MDC/CCL22) and Its Functions

### 2.1. Characteristics of MDC/CCL22

Macrophage-derived chemokine belongs to the CC family of chemokines, meaning there are two cojoined cysteine residues in its structure. Via this classification, it holds the double name MDC/CCL22. MDC/CCL22 is produced by macrophages and dendritic cells, with macrophage-derived chemokine (or MDC/CCL22) belonging to the CC family of chemokines, meaning that there are two cojoined cysteine residues in its structure. MDC/CCL22 is mostly secreted by antigen-presenting cells, i.e., macrophages and dendritic cells, with or without external stimuli. Such stimuli may include bacteria, viruses and parasites, and associated substances, such as lipopolysaccharides, peptidoglycans, CpG motifs, flagella, and viral nucleic acids [11]. The gene for MDC/CCL22 is located in the long arm of human chromosome 16 in a cluster with genes associated with chemokines Fractalkine/CX3CL1 and TARC/CCL17; it consists of 69 aminoacids, but there is not much information on its structure and conformation, besides the fact that its structure was predicted via protein prediction tools such as Alphafold [12,13].

In immune responses, MDC/CCL22 is a potent chemoattractant for CD4 and CD8 T cells, IL-2–activated natural killer cells, as well as for DCs expressing the CCL22 receptor CCR4. The CCR4 receptor is a chemokine receptor that plays a crucial role in the regulation of immune responses and inflammation. Its expression is predominantly observed in various immune cells, including T-lymphocytes, basophils, and platelets. Additionally, it is also expressed in other immune cells, such as monocytes, macrophages, IL-2-activated natural killers, and skin memory T cells. CCR4 receptor expression has also been detected in the endothelial cells of cerebral vessels and coronary arteries, indicating its potential role in the regulation of vascular inflammation.

It is involved in chronic inflammation mediated by the continuous homing of DCs and lymphocytes [14]. Functional activity of MDC/CCL22 is bound to the CCR4 receptor, which is often presented on Th2 cells. This receptor on helper mediates cellular growth and maturation on T-helper subpopulation of T lymphocytes. Therefore, Th1/Th2 polarization is partially mediated by MDC/CCL22. MDC/CCL22 is also involved in cellular migration within thymus. Experiments show that MDC/CCL22 is mostly produced by maturing DCs and activated macrophagic cells [14]. In vivo experiments have demonstrated the constitutive production of MDC/CCL22 by epithelial cells in human thymic medulla [15] and interdigitating DC within T-cell zones of mouse lymph nodes [16].

By its structure, macrophage-derived chemokine is not homologous to most chemokines. It is most similar to thymus- and activation-regulated chemokine (TARC), with a 37% identity [17], which explains its active participation in thymocyte migration. Its role is dual: it is important for adequate lymphocyte development and it aids T regular lymphocytes in their contacts with APCs. TARC/CCL17 is one of the ligands for CCR4 receptor (also known as CD194), along with CCL2 (MCP-1), CCL4 (MIP-1), and CCL5 (RANTES). All CCR4 ligand chemokines are shown to be involved in COVID-19 associated immunity; however, it is more common for all of them, except RANTES/CCL5 and MDC/CCL22, to show elevated concentrations in blood plasma [17,18,19].

The regulation of MDC/CCL22 concentrations is a complex and two-sided process. On one hand, there is upregulation of blood plasma concentrations via Th1 cytokines, such as IL-4 and IL-5. These cytokines stimulate the production and release of MDC/CCL22 from various immune cells. On the other hand, there is downregulation of MDC/CCL22 via interferons, which can inhibit the expression and secretion of MDC/CCL22, resulting in decreased levels in the bloodstream [20]. This two-sided regulation of MDC/CCL22 highlights the intricate and dynamic interactions between different components of the immune system, and it underscores the importance of maintaining a delicate balance between pro-inflammatory and anti-inflammatory responses.

### 2.2. Changes in MDC/CCL22 Concentrations in COVID-19 In Vitro and In Vivo

Adequate regulation of the immune response is the key factor in disease severity. As COVID-19 progresses, it is often accompanied by immunity dysregulation from both cellular and humoral links of the immune responses. Therefore, studying regulatory molecules (cytokines, chemokines) included in this process is vital for understanding COVID-19 immunity.

Within our work, we have studied both cytokines and chemokines in COVID-19, and we also took different approaches to experimental design and data analysis. As the pandemic progressed, newer points of interest emerged: for instance, we investigated the value of cytokine profiling in acute infection and persisting patterns in convalescent patients [21]. We also explored predictors of disease severity among cytokines/chemokines [22], and we suggested a model for prognosis of the disease outcome. However, as the virus changed, we have realized that the immunological landscape went through certain changes as well. Therefore, our study group decided to pay closer attention to the role of viral genetics in COVID-19 immunity [23].

Among the biological substances highlighted in our previous studies (CCL2/MCP-1, CCL3/MIP-1α, CCL4/MIP-1β, CCL7/MCP-3, CCL11/Eotaxin, CCL22/MDC, CXCL1/GROα, CXCL8/IL-8, CXCL9/MIG, CXCL10/IP-10, and CX3CL1/Fractalkine, IL-1α, IL-1β, IL-2, IL-3, IL-4, IL-5, IL-6, IL-7, IL-9, IL-12 (p40), IL-12 (p70), IL-13, IL-15, IL-17A/CTLA8, IL-18, IL-22, IL-27, IFNα2, IFNγ, TNFα, TNFβ/Lymphotoxin-α (LTA), IL-1ra, IL-10, EGF, FGF-2/FGF-basic, Flt3 Ligand, G-CSF, M-CSF, GM-CSF, PDGF-AA, PDGF-AB/BB, TGF-α, and VEGF-A), the most prominent and unexpected role belongs to one of the CC chemokines—macrophage-derived chemokine (MDC/CCL22). Specifically, significantly lower MDC/CCL22 concentrations were seen in COVID-19 patient plasma, independent of SARS-CoV-2 genetic variant (the original Wuhan strain, or the Alpha, Delta, or Omicron variant). This finding was especially interesting in comparison to other chemokines, as their concentrations showed a tendency to rise in EDTA plasma collected from COVID-19 patients in comparison with healthy donors (HD).

Research on MDC/CCL22 in COVID-19 is limited, but a summary of existing studies on MDC/CCL22 in patients with acute COVID-19 and convalescents is presented in Table 1.

At the early stages of the pandemic, when we studied patterns of cytokine profiling in acute phase patients and convalescents, we discovered a noteworthy tendency concerning MDC/CCL22 levels. Convalescents of the original SARS-CoV-2 viral strain (here we address it as the Wuhan strain) had significantly lower MDC/CCL22 concentrations, not only compared to healthy controls but also in comparison with those in the acute stage of the infectious process.

This finding suggests that the impact of the virus on the immune system may be more profound than previously thought. It is unclear whether this decrease is solely due to the virus’s effects on DCs. However, recent studies indicate that MDC/CCL22 may be crucial for regulating immune responses and preventing excessive inflammation by recruiting regulatory T cells. Thus, the depletion of MDC/CCL22 in COVID-19 patients may contribute to immune imbalance and more severe manifestations of the lung inflammation. Further research is necessary to fully comprehend the complex interactions between MDC/CCL22, its producer cells, immune regulation in COVID-19, and coagulation factors.

The abovementioned concentration dynamics can be explained by a possible “depletion” of MDC/CCL22 production, even in the next few months after recovery. It is worth mentioning that, in other studies concerning other inflammation prone illnesses, MDC/CCL22 rarely shows such a decrease, even in diseases affecting the respiratory tract. Therefore, such consistently lower concentrations may be specific to COVID-19 and require thorough investigation.

Our previous studies showed that COVID-19 recovery is often associated with decreases in several cytokines, with MDC/CCL22 being only one of them [24,25,26]. Such a depletion and dysregulation in the cytokine branch has been described by other researchers in works concerning the first 8 months since the recovery [27,28,29]. At the same time, we noted changes affecting cellular immunity [28] even more than 6 months after recovery: for instance, decrease in CD8+ effector subsets and higher numbers of CD8+ T cell subsets associated with homing molecules (Tc2, Tc17, Tc17.1) within the lung tissue and mucosal tissues. This finding was confirmed by other researchers [30,31]. It therefore proves that COVID-19 has a certain effect on immune cells and their mediators even after recovery.

**Table 1:** Summary of research on COVID-19 focusing on the levels of MDC/CCL22 in the blood plasma of patients in the acute phase and those who have recovered. “N.A.” marks the parameters not applicable for the study in question.

Study	MDC (Me) in COVID-19 Patients	MDC (Me) in COVID-19 Convalescents	MDC (Me) in Healthy Donors	COVID-19 vs. Healthy Donors (*p*-Value)	COVID-19 vs. Convalescents (*p*-Value)	Convalescents vs. Healthy Donors (*p*-Value)
Arsentieva et al. [21]	872.7 pg/mL	653.5 pg/mL	1155.0 pg/mL	N.A.	*p* < 0.05	*p* < 0.0001
Arsentieva et al. [22]	254.0 pg/mL for survivors 230.7 pg/mL for non-survivors	557.3 pg/mL	N.A.	*p* < 0.05 for survivors *p* < 0.01 for non survivors	N.A.	N.A.
Korobova et al. [23]	629.8 pg/mL for Wuhan strain 474.1 pg/mL for Alpha variant 344.1 pg/mL for Delta variant 306.1 pg/mL for Omicron variant	N.A.	N.A.	*p* = 0.0005 for Wuhan strain *p* = 0.0067 for Alpha variant *p* < 0.0001 for Delta variant *p* < 0.0001 for Omicron variant	N.A.	N.A.
Tufa et al. [28]	438.9 ng/L	N.A.	725.7 ng/L	*p* < 0.0001	N.A.	N.A.
Ling et al. [29]	Mild—724.9; Moderate—495.44; Critical—399.2 pg/mL	Mild—644.8; Moderate—6483; Critical—220.3 pg/mL	N.A.	N.A.	*p* ≤ 0.001 (for critical)	N.A.

In the study by Ling et al., a statistically significant decrease in MDC/CCL22 was noted not only during acute COVID-19 but also a year after the infection [29]. This data overlaps with our own findings: in convalescent patients, we noted a statistically significant decrease in MDC/CCL22 between 30 and 100 days since the onset of the disease. Tufa et al. confirmed our finding concerning lower levels of MDC/CCL22 in patients with COVID-19, as their study highlighted the same tendency [28], and it is noteworthy that they reported negative correlation between MDC/CCL22 and the course of COVID-19.

Recent studies have highlighted the well-described phenomenon of persistent COVID-associated lymphopenia depletion. This phenomenon has been reported to persist even after patients have recovered from the virus [30]. Multiple studies of MDC/CCL22 role in inflammatory regulation have highlighted the importance of this chemokine [9,30,31]. Its presence contributed to the regulatory T cell activation and constrained inflammatory processes within type I helper T cells as it is shifting the immune responses towards Th2.

Such findings provide grounds for discussing changes in immunity both in acute phase infections and in COVID-19 convalescents. In 2022, the International Statistical Classification of Diseases and Related Health Problems (ICD) registered a new diagnosis of “post-COVID syndrome”, which included a wide spectrum of symptoms noticeable long after COVID-19 recovery [32]. These symptoms include fatigue, shortness of breath, a chronic cough, muscle and joint pains, heart palpitations, persistent vascular changes, i.e., recurrent thrombosis, and mental health issues (anxiety, depression, and memory and concentration loss). The reasons behind these symptoms are yet to be investigated.

Studying post-COVID is a new challenge brought by the pandemic, and this challenge has yet to be overcome by global scientific efforts. As the consequences and complications of this novel phenomenon are being discovered, the mechanisms behind post-COVID are still unknown. Even now, however, it is clear that cytokines (and chemokines) play a pivotal role in the immune responses behind this process. Keeping in mind the knowledge already collected and described in the past, we may presume that MDC/CCL22 plays a bigger role in post-COVID than is currently understood.

### 2.3. Macrophage-Derived Chemokine MDC/CCL22 in Various Pathologies

#### 2.3.1. MDC/CCL22 in Oncology of Non-Respiratory Organs

Novel areas of research in oncology provide the most thorough insight into the role of MDC/CCL22 in the disease development. Macriophage-derived chemokine is a potential biomarker of antigen-presenting cells activity and of immune suppression. It reflects the qualitative and quantitative properties of anti-tumor responses in the host. Such responses are usually negatively affected, either by the escape mechanisms imprinted in tumor cells or by immunosuppressive treatment in oncology.

Increased concentrations of MDC/CCL22 in transformed tissues has been shown to lead to T regulatory cells infiltration in melanoma as well as ovarian, prostate, or breast carcinomas [33,34,35]. Circulating MDC/CCL22 levels are related to both glioma risk and survival duration independent of age, histology, grade, and IDH mutation status. CCL22 should be considered a marker of immune status with potential prognostic value [36]. In contrast, it was shown that tumors that showed normal or suppressed MDC/CCL22 expression were not infiltrated by Tregs, regardless of whether they produced other CCR4-binding chemokines, including other potent agent of T-cellular responses, TARC/CCL17 [37].

As shown in tumor models, MDC/CCL22 is vital for recruitment of intratumoral T regulatory cells [38].

MDC/CCL22 is a chemokine that has been extensively studied in the field of oncology as a potential target for monoclonal antibody therapy. This protein is known to have a part in the recruitment of immune cells to the tumor site, and blocking its activity may represent a promising strategy for treating cancer. However, despite its importance in cancer biology, the precise function of MDC/CCL22 in the immune system remains poorly understood. Researchers are actively working to unravel the complex signaling pathways and interactions that underlie this protein’s activity, with the goal of developing new therapeutic approaches to a range of diseases.

#### 2.3.2. Macrophage-Derived Chemokine MDC/CCL22 in Autoimmunity and Atopic Diseases

As is the case with many other respiratory infections, COVID-19 is followed by extensive inflammatory reactions. These inflammatory reactions, however, can affect not only the lung tissue but, actually, anywhere in the body, as long as SARS-CoV-2 can find a potential target for binding.

Therefore, it is important to study how MDC/CCL22 acts in other pathologies followed by inflammation. For instance, autoimmune and atopic diseases are characterized by the inadequate involvement of immune cells to the inflammatory process. In such cases, there is a potent production of pro-inflammatory markers. In the studies concerning MDC/CCL22 in autoimmune diseases and atopic dermatitis (AD), it shows prominent increase. For instance, MDC/CCL22 is usually overexpressed in the lesioned skin of atopic dermatitis (AD). IL-4 and IL-13 expression in Th2 cells in lesions affected by AD is more potent than unaffected Th2 cells. Dendritic cells respond to IL-4 and IL-13 by secreting MDC/CCL22 (as well as TARC/CCL17 and MIP-4/CCL18) [39]. Plasma concentrations of MDC/CCL22 and TARC/CCL17 are significantly increased in patients with AD compared to those in healthy controls, and they are associated with disease severity in AD.

Interestingly, MDC/CCL22 has also been found to play a role in other inflammatory conditions. For example, MDC/CCL22-producing resident macrophages have been associated with the inflammatory profile of patients with autoimmune Sjögren’s Syndrome [40], an autoimmune disease affecting the secretory glands in the body.

In vitro models of autoimmune encephalitis show increased levels of MDC/CCL22, but in this context, MDC/CCL22 mediates chronic inflammatory processes by recruiting T regulatory cells to glia [41]. This suggests that MDC/CCL22 may have a broader role in modulating immune responses than simply intensifying inflammation.

#### 2.3.3. Macrophage-Derived Chemokine MDC/CCL22 in Respiratory Disease

Since coronavirus infection is primarily accompanied by damage to the upper and lower respiratory tract, the role of MDC/CCL22 in the development of pulmonary pathology is of interest. In tuberculosis, MDC/CCL22 shows lower concentrations in vitro in cells and autologous platelets taken from patients infected with *Mycobacterium tuberculosis*. It is known, however, that COVID-19 is often accompanied by thrombosis and elevation of platelet-derived factors [42].

Nakanishi et al. showed that in patients with lung cancer (adenocarcinoma, squamous cell carcinoma), high levels of MDC/CCL22 are associated with less complications in the post-surgical period. Moreover, MDC/CCL22 levels reflected a negative correlation with the risk of recurrent malignancy [43]. In the post-surgery period, patients with malignant tumors in the lung show higher survival rates when the immune cells infiltrate the lung tissue. In such cases, MDC/CCL22 acts as a mediator and a regulator for recruitment of the cells to the lung tissue.

In murine models, it has been shown that i.v. introduction of recombinant MDC can enhance inflammation in lung tissue [44]. One of the possible explanations is an intensive mobilization of certain immune cells: macrophages and type II helper T cells. Additional work performed by the same group demonstrated that the blockage of NFκB signaling leads to a decrease in MDC/CCL22 gene expression in response to proinflammatory stimuli [39]. In the post-surgical period, patients with malignant lung tumors show higher survival rates when immune cells infiltrate lung tissue [45]. In such cases, MDC/CCL22 acts as a mediator and a regulator for recruitment of cells to the lung tissue.

In most cases, when MDC/CCL22 levels are compared between healthy individuals and those with other illnesses, such as cancer or autoimmune diseases, a significant increase is typically observed. However, in the case of COVID-19, the opposite trend is witnessed as MDC/CCL22 levels decrease. In the following sections, we will explore potential reasons for this phenomenon.

### 2.4. Possible Mechanisms behind the Decrease in MDC/CCL22 Concentrations in COVID-19

**Figure 1:**
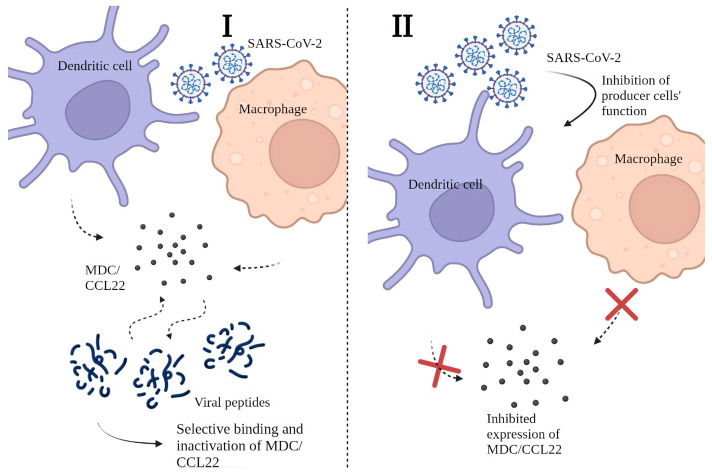
Possible mechanisms for lower MDC/CCL22 concentrations in COVID-19 patients’ plasma: (**I**) Decrease in MDC/CCL22 concentration associated with selective binding to SARS-CoV-2 viral peptides; (**II**) Restriction of MDC/CCL22 secretion by producer cells due to their functional failure.

We believe that the decrease in MDC/CCL22 in COVID-19 patients’ plasma is note-worthy. Since the reasons behind this phenomenon are not yet clear, we present hypothetical explanations for the phenomenon in question in Figure 1.

Our hypothesis implies two possible mechanisms for lower MDC/CCL22 concentrations in COVID-19.

It is possible that SARS-CoV-2 viral proteins are capable of binding MDC/CCL22 due to potential affinity with, or mimicry of, MDC/CCL22’s main ligands. Thus, MDC/CCL22 production by producer cells (i.e., DCs and macrophages) is unperturbed. Yet, the selective binding of this chemokine makes it undetectable for commercial kits as its binding sites or antigenic structure become unavailable. Moreover, for the same reason, there is a possibility of reduction of MDC/CCL22 functional activity. This hypothesis can be supported by the fact that other biological substances (e.g., cytokines) produced by APCs, show increased concentrations in COVID-19 patients when compared to healthy donors [9,10,11,14,25,46]. The infectious agent requires mechanisms of evading the immune responses, and one of the potential ways is to block chemoattraction by inactivating chemokines [47]. Although the concept of chemokine binding proteins is not entirely new, the nature of these peptides or proteins and their specific properties are yet to be discovered, and the whole concept of protein-based binding in COVID-19 requires in vitro experiments. Proteomics studies concerning cytokine dysregulation in the presence of SARS-CoV-2 proteins have been previously performed [48], yet there is no information covering potential interactions between MDC/CCL2 and viral proteins. Among known proteins, ORF (open reading frame) 8 is known to bind with the dendritic cells and alternate cytokine expression [49]. At the same time, the S protein of SARS-CoV-2 was shown to play a part in the activation of several proinflammatory cytokines [50]. Within the same study, membrane (M), envelope (E), and nucleocapsid (N) proteins did not trigger inflammatory responses as potent as the one caused by the S protein. However, the S protein is known to be less conservative [51]: it undergoes changes in its structure with each genetic change in the virion proteins’ structure. The N-protein structure is believed to be more stable, and as MDC/CCL22 shows stable concentrations independent of the genetic variant, it is more likely to be a target for MDC/CCL22 binding. In the study by López-Muñoz, it is shown that the N-protein can form a bond with chemokines through its GAG-binding domain. It is known to inhibit chemokine-mediated leukocyte migration in vitro [52]. In other viral infections, such binding of chemokines to viral proteins is well known and previously described [53].

We also suggest another potential mechanism for MDC/CCL22 depletion. It is possible that COVID-19 can actually affect the functional activity of macrophage-derived chemokine producer cells. In previous studies, it was highlighted that COVID-19 patients often demonstrate a significant shortage of DCs. This tendency was observed both in acute and post-recovery periods [54,55,56]. Such a shortage is seen not only in a quantitative but also in a qualitative way––specifically, in vitro studies showed depletion of functional activity in dendritic cells [57]. Moreover, other studies have demonstrated correlations between the severity of clinical symptoms and DCs as a population. However, cytokines and chemokines produced by DCs seem to be unaffected by the depletion of these cells or their functional inability. These cytokines include IL-1α, IL-1β, IL-6, IL-7, IL-12 (p35 and p40), IL-15, IL-18, TNF-α, TGF-β, macrophage CSF, and granulocyte-macrophage CSF; their concentrations tend to increase in COVID-19 [58]. In our studies, some of these cytokines showed a typical pro-inflammatory profile and an actual increase in concentrations [10,11,12]. Other studies exploring the role of DCs in coronavirus infections highlight their participation in viral dissemination [59]. Notably, not only in SARS-CoV-2 associated diseases but also in SARS and MERS [60], DCs are named “the missing link” between antiviral innate and adaptive responses. As SARS-CoV-2 induces activation of specific plasmacytoid IFN-producing cells (pDCs) [61], it may be possible that this subpopulation of DCs displaces conventional DCs (cDCs) and monocyte-derived DCs (MoDCs) [62,63]. Plasmacytoid dendritic cells are characterized by CD123+CD303+ phenotype, and their main role is enabling type I IFN expression. Moreover, the severity of the disease correlates with the responsiveness of pDCs [64,65]. Although there is little information on the ability of pDCs to produce MDC/CCL22, there is a possibility that it is not as prominent as in other types of dendritic cells (e.g., MoDCs). If this assumption is correct and pDCs’ production of MDC/CCL22 is in any way impaired, this may explain the differences in chemokine production associated with the coronavirus infection.

Theoretically, MDC/CCL22 depletion in COVID-19 may be explained by the abovementioned phenomenon. Although the specific mechanisms behind DC functional suppression by COVID-19 are not described, there is the possibility that this chemokine holds a more important role than was previously believed. Further research is required to fully investigate the complex interactions between the virus, DCs, and MDC/CCL22. Any findings on the topic can be illuminating in terms of new therapeutic strategies to combat COVID-19 and other infectious diseases. We encourage researchers to join the conversation.

**Figure 2:**
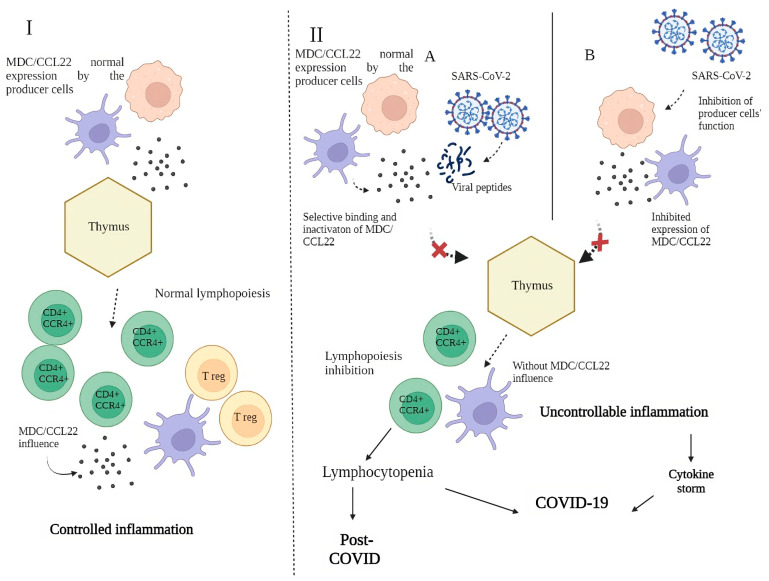
Role of MDC/CCL22 in immunity and the SARS-CoV-2 infectious process. (**I**) MDC/CCL22 influence on T lymphocyte maturation in thymus via the CCR4 receptor. The presence of this chemokine also mediates an adequate balance between regulatory T cells and helper T cells, thus creating restrictions on inflammatory reactions. (**II**) SARS-CoV-2 influence on T cell maturation in thymus via depletion of MDC/CCL22: (**A**) decrease in MDC/CCL22 concentrations associated with selective binding to SARS-CoV-2 viral peptides; (**B**) restriction of MDC/CCL22 secretion by producer cells due to their functional failure.

Both proposed mechanisms prove that SARS-CoV-2 plays a certain role in the suppression of DCs. Potential mechanisms for inflammatory dysfunction in patients with lower MDC/CCL22 are presented in Figure 2.

## 3. Conclusions

As explained earlier, MDC/CCL22 plays a mediating role for both Th2 immune responses and for regulatory T cells. Presumably, in the absence of this chemokine, inflammatory responses might shift towards hyperergic. Long-term depletion of MDC/CCL22 concentrations in blood plasma of acute COVID-19 patients and convalescents may explain the relatively greater severity of COVID-19 in comparison with other respiratory viral infections.

Keeping in mind preexisting data on several related topics (macrophage-derived chemokine, COVID-19, and concomitant hyperinflammatory profiles), we have grounds to presume that MDC/CCL22 may be a missing link in the chain of as yet unexplained processes in COVID-19. It is important to consider its role in vaccine-associated immunity, especially in individuals who have previously undergone severe COVID-19. Taking into consideration the fact that MDC/CCL22 concentrations drop in COVID-19, a hyperinflammatory profile and inadequate reaction to external stimuli may become potential problems in immunity formation.

Due to the abovementioned features of coronavirus infection, the phenomenon of MDC/CCL22 still demands further research.

## Data Availability

Data sharing not applicable.
